# Utility of 4D CT in endoleak characterization after advanced endovascular aortic repair

**DOI:** 10.1177/17085381221105326

**Published:** 2022-06-02

**Authors:** Massimo Tarulli, Kong Teng Tan, Thomas Lindsay, Daniyal Nasir Mahmood, Sam Santiago, Arash Jaberi, Sebastian Mafeld

**Affiliations:** 1 7989 University Health Network and Mount Sinai Hospital, Medical Imaging, Toronto, ON, Canada; 2 4257 Queen’s University, Kingston, ON, Canada

**Keywords:** Endovascular, endoleak, 4D, 4D CT, branched, fenestrated

## Abstract

**Objectives:**

To assess the performance of dynamic or 4D CT in characterizing endoleaks in advanced endovascular aortic repair (branched and fenestrated) when other modalities fail to fully characterize the leak, most often conventional CTA.

**Methods:**

Retrospective review of 13 patients from 2008 to 2021 who underwent 16 4D CTs to characterize endoleaks in branched and fenestrated endovascular aortic repair (FB-EVAR). The 4D CTs were performed covering up to 16 cm of the *z-axis*, with anywhere between 10 and 40 iterations performed every 2 s. These settings were adjusted depending on graft characteristics and type of endoleak suspected. The scans were assessed for their ability to detect the endoleak (sensitivity), and further to characterize the endoleak by type and subtype (specificity).

**Results:**

Overall sensitivity in 16 scans for endoleak detection was 100%. There was a specificity of 87.5% for determining the type of endoleak (14/16). These results included two studies that were inconclusive and repeated due to technical difficulties. In patients where a specific subtype was not established, the leak was localized to the appropriate target vessel. Average dose for the 4D CT was 4724 mGy*cm (1108–11069), with the outlining higher dose scans secondary to higher iterations in those scans.

**Conclusions:**

4D CT is a useful adjunctive tool in FB-EVAR surveillance with excellent sensitivity and specificity in characterizing endoleaks. This allows for accurate localization of leaks, which is critical for management planning.

## Introduction

Endovascular repair of thoracic and abdominal aortic aneurysms has become more common, demonstrating similar long-term outcomes with improved perioperative morbidity and mortality, and decreased hospital stay compared to open repair.^1,2^ Accordingly, fenestrated and branched endoprostheses are being used more frequently for complex aortic anatomy. Persistent perfusion of the aneurysm sac (or endoleak) is the most common complication of endovascular aortic aneurysm repair.^3^ Table 1 summarizes the different types of endoleaks. With the increasing incidence of FB-EVAR and increasing complexity with multiple graft/branch/fenestration interfaces, new subtypes of endoleak (Type 1 and 3) for target vessels have been included.^4^

**Table 1.** Endoleak classification for fenestrated endovascular aortic repair.

Type	Subtype	Description
1	A	Proximal graft attachment site perfusion
	B	Distal graft attachment site perfusion
	C	Side branch attachment site perfusion
2		Retrograde perfusion of aneurysm via aortic branches
3	A	Junctional leak between main body graft components
	B	Fabric tear or fracture
	C	Junctional leak between target vessel components
4		Graft porosity
5		Endotension with aneurysm growth without discernable imaging findings of an endoleak

Accurate detection of endoleaks is crucial in determining longer term management strategies; however, characterization of endoleaks is more challenging in fenestrated and branched endovascular aortic repair (FB-EVAR) with multiple potential sources as described above.

Imaging modalities for surveillance after aortic endovascular repair include plain radiography, ultrasound (with or without contrast), CT angiography (classically unenhanced, arterial and delayed phase images), MR angiography, and conventional angiography.^5,6^ CT angiography is the mainstay of surveillance as images are easily reproducible, and the aorta and branches are very well visualized.^7^ Conventional triphasic CT provides high sensitivity and specificity for endoleak detection, approximately 85% and 95%, respectively.^8^ Ultrasound is an alternative surveillance option and can be augmented with contrast enhanced ultrasound (CEUS); however, operator dependence, availability, and identifying/interrogating branches/fenestrations can be challenging. Therefore, in circumstances of difficult to detect endoleaks, CEUS can be a good screening tool, but the traditional “gold standard” has been triphasic CT. Conventional angiography can be used, and treatment can be offered in the same session; however, in complex branched/fenestrated cases, interrogating different endoleak sources can be very challenging and often requires different vascular access routes for diverse angles of visceral branches. It is important therefore to establish the specific endoleak location and type to best guide therapy and minimize the risk of therapy-related complications. The timing of contrast enhanced phases in conventional CTA provides only two discrete time points for assessment. Although this is sufficient to detect and characterize many endoleaks, the exact type of early and large leaks may remain ambiguous. Further, the location of endoleak can be nebulous in complex branched/fenestrated repairs with close proximity of the visceral branches to each other. For these reasons, dynamic (or four dimensional) CTA was proposed to provide the ability to follow the contrast bolus in a limited field of view at multiple time points (often a few seconds apart), which has allowed better specificity for endoleak type and location in diagnostically challenging situations.^9^ It has been shown to provide excellent specificity in cases of difficult to diagnose endoleaks and even decreased overall radiation and contrast load when re-intervention is eventually performed.^10^ To our knowledge, there are no specific studies that have evaluated the performance of 4D CT specifically for complex branched/fenestrated repairs.

The purpose of this study was to summarize our experience with the use of 4D or dynamic CTA for assessing endoleaks, with a specific focus on those performed in complex aortic endovascular repairs where the type and/or source of leak was uncertain using other methods of assessment.

## Materials and methods

This retrospective study was approved by the Institutional Review Board at University Health Network. Patients who underwent complex branched and/or fenestrated endovascular repair between 2008 and 2021 were reviewed (264 patients). Cross referencing was performed against a Montage™ database search of radiological reports containing the keywords “4D,” “dynamic,” and “endoleak.” All patients who underwent a 4D CT were included. Patients for whom 4D CT was recommended and not performed, and patients who underwent 4D CT for standard EVAR were excluded.

## 4D CT technique

An Aquilion One Prism (Canon Medical Systems Corporation, Otawara, Tochigi, Japan) 320 slice scanner was used to acquire up to 16 cm field of view in the *z-axis*. An unenhanced scan was first performed. Subsequently, a timing bolus with 20 cc of Visipaque 320 or Ultravist 300 was administered at 5cc/sec to detect contrast at the top of the graft in the field of view (10 second delay typically). Once the appropriate start time was established, the larger contrast bolus (70–160 cc) was administered at 5cc/sec. At the previously obtained optimal start time, intermittent scanning was performed, with each acquisition at 100 kV/350 mA with 1 mm thick slices (0.5 s per acquisition). The acquisitions were performed every 2 s until 65 s (or longer if needed). A 2 min delayed scan was also performed. 3D post-processing was performed. See Table 2 for a summary of technical details.

**Table 2.** Standard dynamic CTA specifications.

Parameter	Value
Acquisition iterations	Variable (10–40)
Tube voltage (kVp)	120 (AP and lateral)
Tube current (mAs)	50 (AP) 100 (lateral)
Rotation time (s)	0.5
Scan time (s)	0.5
Resolution time, s	2
Total scan time, s	120
Dose length product (DLP) (mGy·cm)	4724 (1108–11069)
Scan range (*z-axis*, cm)	Up to 16
Slice thickness (mm)	1
Contrast material volume (mL)	112 average (70–160)
Contrast flow rate (mL/s)	4–5

Post-processing was completed on the Aquilion One console and was also transferred to a dedicated post-processing workstation for creation of 4D digital subtraction CTA images as well as 3D and thin-section maximum-intensity projection cine loops.

The images and reports were reviewed using the Coral PACS system (as well as reconstruction algorithms) and the clinical history for the aforementioned patients was reviewed to establish if additional intervention was performed, as well as the outcome.

## Results

The imaging reports were queried with the Montage™ search tool, using the keywords “4d,” “dynamic,” and “endoleak.” Combined results yielded 46 individual entries. CTA reports that suggested 4D or dynamic CT were excluded and standard EVAR patients who underwent 4D CT were also excluded. Thirteen patients with 16 4D CTs performed were included in the retrospective review cohort. Patient characteristics are summarized in Table 3.

**Table 3.** Patient characteristics.

Characteristic	Value
Age	72.4 +/− 7.8
Sex	5 female, 8 male
BMI	28.5 +/− 5.2
eGFR	67 +/− 22.8
Hypertension	9/13
Hyperlipidemia	6/13
Peripheral vascular disease	3/13
Diabetes	2/13
Coronary artery disease	3/13
COPD	5/13
Smoking history	9/13

The endovascular aortic repairs performed in these 13 patients had a mean of 1.6 branches and 2.1 fenestrations.

A summary of the 4D CT results is in Table 4 (first 4D CT) and 5 (second 4D CT). The first 4D CT was performed a mean of 270.5 days post aortic repair (ranging from 2 days post index procedure to 835 days) with 3 patients receiving a 4D CT within 10 days of the index procedure. The most common reason for 4D CT was detection of an endoleak in conventional CTA without a definite type or source identified (10/13). 4D CT was used in 2/13 patients for persistent endoleak despite previous treatment. The most common investigation before the 4D CT was conventional CTA (11/13). Contrast enhanced ultrasound (CEUS) and angiography was performed in the other 2/13 patients before 4D CT. 4D CT was able to detect/confirm endoleak in 100% of patients. The endoleak was successfully characterized in 11/13 patients. After the first 4D CT 7/13 patients underwent re-intervention to repair the leak.

**Table 4.** 4D CT details and results.

Patient	Time to scan (days)	History	Previous imaging	Suspected endoleak type(s)	Endoleak detected?	Type	DLP (mGy cm)
1	100	Endoleak NYD	CTA	3	Yes	2	5522.6
2	835	Persistent endoleak despite treatment	CTA	2, 3	Yes	3c	5715.1
3	4	Endoleak NYD	Angiogram, CTA	1, 3	Yes	3c	8847.6
4	9	Endoleak NYD	Angiogram, CTA	2, 3	Yes	2	6293.5
5	415	Endoleak NYD	Angiogram, CTA	2	Yes	3b or c	1508.4
6	313	Endoleak NYD	Angiogram	3 (subtype uncertain)	Yes	Uncertain	1544.5
7	310	Endoleak NYD	CEUS	3	Yes	3a or b	3381.7
8	222	Endoleak NYD	CTA	3 (subtype uncertain)	Yes	3c	11689.7
9	118	Endoleak NYD	CTA	3 (subtype uncertain)	Yes	3c	8956.6
10	683	Endoleak NYD	CTA	3 (subtype uncertain)	Yes	3c	4242.6
11	83	Endoleak NYD	Angiogram, CTA	Uncertain proximal, type 2 distal	Yes	Uncertain	1149.6
12	2	Type B dissection post EVAR	CTA	1A	Yes	1a	1108.8
13	422	Persistent endoleak despite treatment	CTA	3	Yes	3c	1461.5

Three patients underwent an additional 4D CT. One was performed for a denovo leak approximately 2 years after the first 4D CT, and the other two 4D CTs were repeated to rectify technical difficulties in the first 4D CT (one day after the index scan). Table 5

**Table 5.** Patients with additional 4D CT.

Patient	Time to scan (days)	History	Previous imaging	Suspected endoleak type(s)	Endoleak detected?	Type	DLP (mGy cm)
1	634	Endoleak NYD	CTA	Uncertain	No (resolved)	NA	2290.8
6	1	Persistent endoleak despite treatment	CTA	3 (subtype uncertain)	Yes	3b or c	2295.9
11	1	Endoleak NYD	Angiogram, CTA	Proximal leak uncertain, type 2 distal	Yes	3a	1340.9

Two patients with additional 4DCT underwent subsequent re-intervention. One patient in this cohort had an endoleak that resolved spontaneously. Therefore, no endoleak was detected in the 4D CT.

Including the two repeated 4D CTs due to technical issues, the overall sensitivity was 100% for detecting endoleak (i.e., the 4D CT was able to confirm the presence of an endoleak) and specificity of 87.5% for determining the specific type of endoleak (14/16). Not all scans of patients with Type 1 and 3 endoleaks (13 scans) were able to establish the correct subtype, 8/13 (61.5%). Despite this, it is important to note that 2 of these unsuccessful scans were those associated with technical difficulties. Also, the remaining 3 scans were able to localize the leak to a specific branch, which effectively guided management.

**Figure 1.**
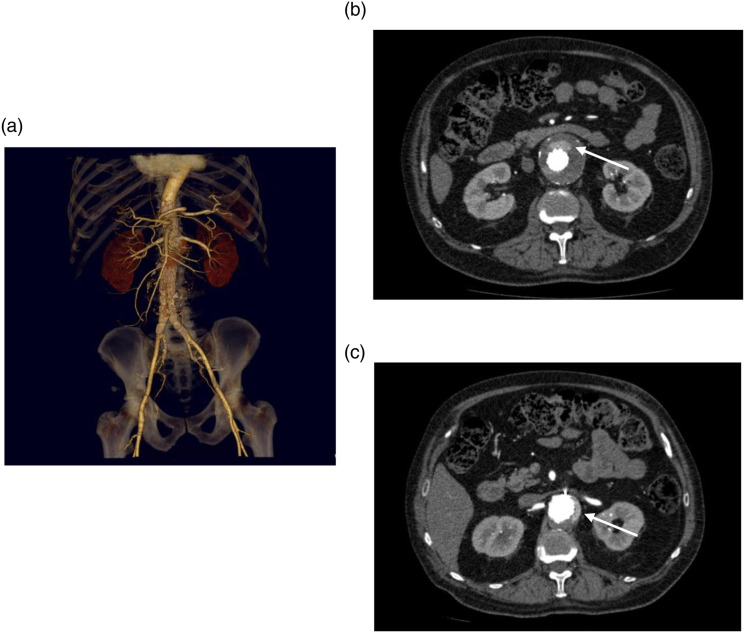
(a) Reconstruction of conventional CTA for 4 fenestrated endograft. (b and c) demonstrate representative axial images of the CTA with endoleak of unclear origin within the anterior and left aneurysm sac (white arrows).

**Figure 2.**
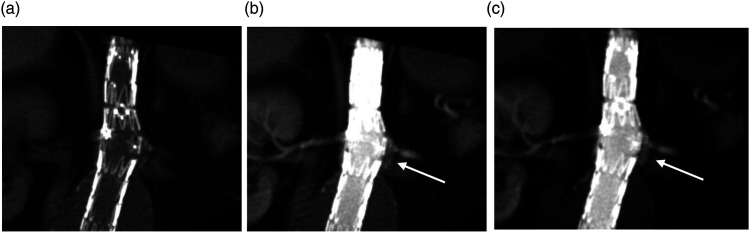
4D CT with unenhanced (a) and early contrast enhanced phases (b and c) demonstrating early contrast enhancement below the left renal stent consistent with a Type 3c leak (white arrows).

A typical sequence of imaging for a patient with a difficult to diagnose/characterize endoleak can be seen in Figures 1–2. Figure 1 demonstrates conventional CTA for a patient with a fenestrated abdominal aortic endograft (4 fenestrations) with an endoleak of unclear origin. Representative axial arterial phase images demonstrate contrast in the aneurysm sac near visceral vessels anterior to the main graft and below the renal fenestration; however, the exact source of the endoleak is unclear. Figure 2 demonstrates 4D CT images which clearly delineate the flow/progression of contrast directly inferior to the left renal fenestration clearly identifying the Type 3c endoleak. Attached video clips of the 4D CT further illustrate the endoleak.

## Discussion

Endoleak is the most common complication after endovascular aortic repair.^3^ These endoleaks can lead to short term aneurysm expansion and risk of rupture. Lifetime surveillance is therefore necessary. CT angiography, typically with three phases (unenhanced, arterial, and delayed) is the mainstay of surveillance with excellent sensitivity for detection of endoleaks. However, in a minority of patients, conventional CTA fails to fully characterize the type of endoleak which is required to guide therapy. In complex fenestrated and/or branched repairs with multiple potential sources of endoleak, identification of the endoleak origin can simplify the treatment access and therapy. Dynamic or 4D CTA was proposed to closely follow the contrast bolus at multiple time points, allowing better characterization and localization of difficult to diagnose endoleaks.^9^ Initial feasibility work also involved optimizing the timing of the dynamic acquisition to maximize contrast differences for different types of endoleak and more recent time-attenuation curve (TAC) or time-to-peak enhancement (Δ TTP) analysis has been proposed as adjunctive strategies in 4D CT interpretation; however, the utility of this fenestrated and branched repair is unknown.^11^ Early case reports have shown potential utility of practical use of 4D CT in endoleak in FB-EVAR.^12^ Finally, dynamic CTA has been shown to provide excellent specificity in cases of difficult to diagnose endoleaks compared to conventional CTA and decreased overall radiation and contrast load when re-intervention was performed in these patients in prospective studies.^10^ Literature to date is summarized in Table 6. To our knowledge, there are no specific studies that have evaluated the performance of 4D CT specifically for complex branched/fenestrated repairs.

**Table 6.** 4D CT for endoleaks, literature summary.

Study	Date	Study description	Patients	Aneursym type	Results
Sommer et al^9^	2013	Prospective, comparing dynamic CT to reference standard CEUS for thoracic and abdominal aortic grafts in high risk patients	*N* = 54	Thoracic (T) 8, Abdominal (A) 46, in which CEUS was also performed	23 leaks detected (4 T, 19 A), SN 94%, SP 93%, PPV 89%, NPV 96%
Lehmkuhl et al^11^	2013	Retrospective review of 4D CTs to analyze optimal timing for endoleak detection	*N* = 72	Abdominal (A)	44 endoleaks, peak enhancement in aneurysm sac very different than aorta, 4D helpful
Bent et al^12^	2010	Case report of 4D CT in complex FB-EVAR	*N* = 1	FB-EVAR	4D CT performed well in delineating a difficult to diagnose endoleak
Apfalterer et al^13^	2021	Prospective study for quantitative analysis of dynamic CTA for endoleak	*N* = 20	Abdominal (A)	Higher diagnostic confidence for endoleak detection in 4D versus conventional CTA
Koike et al^14^	2014	Prospective study for detection of endoleak with low dose/Fowler position 4D CT in post EVAR patients	*N* = 39	Abdominal (A)	Endoleaks in 8 patients, high diagnostic confidence
Hou et al^10^	2019	Prospective randomized study 4D CT vs CTA for difficult to diagnose endoleaks	*N* = 24	Abdominal (A)	4D > CTA for endoleak characterization (100% accuracy for type), less overall radiation and contrast dose

Two patients required an additional 4D CT because of technical issues. In one of these patients the visceral branch responsible for the endoleak was not included in the field of view of the 4D CT, therefore the endoleak was detected however and the exact source was not certain. This was rectified with an additional 4D CT in a different field of view and the endoleak was fully characterized. In the second patient, the endoleak was early and the time delay used was too late to detect the early movement of contrast into the aneurysm sac, therefore specific characterization was not possible. This was rectified with an additional 4D CT and the delay from bolus injection to first acquisition was shorter, allowing detection and characterization of the endoleak. These two cases highlight the importance of planning in 4D CT. The *z-axis* field of view of this modality is limited to 16 cm with a 320 slice scanner. For more extensive branched/fenestrated repairs, coverage of every component of the endoprosthesis may be impossible. Therefore, an index of suspicion to the location of the endoleak is very helpful to guide the appropriate field of view. Appropriate timing of the delay between contrast injection and acquisition is determined after a test bolus, classically with a reference marker placed in the lumen of the graft. If the endoleak is early the contrast may traverse into the aneurysm sac before the lumen of the graft, leading to a time delay that captures the leak relatively late, making the source ambiguous. If an early endoleak is suspected it may be helpful to start intermittent acquisition earlier, although this should be balanced with radiation dose as earlier acquisition would also tend to lead to more iterations and higher radiation dose.

The major drawback of CTA is ionizing radiation, especially considering the need for lifetime surveillance. Conventional CTA is limited to three phases which limits the radiation dose. 4D CT however has considerably more radiation. Also, compared to other studies, radiation doses in our study were higher with significant variation from patient to patient. This is likely due to more iterations in our patient cohort compared to others. Therefore, 4D CT should only be used when necessary and the number of acquisitions performed during a 4D CT should be as few as possible to correctly characterize the endoleak.

Our study is limited to one tertiary care center with a limited number of 4D CTs performed (16 total), despite over 300 branched/fenestrated EVARS performed at this center. This demonstrates efficacy of conventional CTA in the surveillance of endovascular repair. In cases where an endoleak was not fully characterized (or persisted despite repair), 4D CT provided excellent sensitivity and specificity which was critical in planning for re-intervention.

## Conclusion

4D CT is a useful adjunctive imaging modality for challenging or recurrent endoleaks in patients with complex endovascular aortic repairs. Care should be taken in planning the 4D CT to optimize the timing, reduce the radiation dose, and increase likelihood of identifying the source of the leak which guides further management.

## References

[1] Lederle FA Kyriakides TC Stroupe KT , et al. Open versus endovascular repair of abdominal aortic aneurysm. New Engl J Med 2019; 380(22): 2126–2135. 31141634 10.1056/NEJMoa1715955

[2] Patel R Sweeting MJ Powell JT , et al. Endovascular versus open repair of abdominal aortic aneurysm in 15-years’ follow-up of the UK endovascular aneurysm repair trial 1 (EVAR trial 1): a randomised controlled trial. The Lancet 2016; 388(10058): 2366–2374. 10.1016/S0140-6736(16)31135-7 27743617

[3] Zaiem F Almasri J Tello M , et al. A systematic review of surveillance after endovascular aortic repair. J Vasc Surg 2018; 67(1): 320–331, e37. 28662928 10.1016/j.jvs.2017.04.058

[4] Oderich GS Forbes TL Chaer R , et al. Reporting standards for endovascular aortic repair of aneurysms involving the renal-mesenteric arteries. J Vasc Surg 2021; 73(1): 4S–52S. 32615285 10.1016/j.jvs.2020.06.011

[5] Pandey N Litt HI . Surveillance imaging following endovascular aneurysm repair. Semin Intervent Radiol 2015; 32(3): 239–248. 26327742 10.1055/s-0035-1556878 PMC4540620

[6] Kim S Litt H . Surveillance imaging following endovascular aneurysm repair: state of the art. Semin Interv Radiol 2020; 37: 356–364. 10.1055/s-0040-1715882 PMC7540641 33041481

[7] Harky A Khan D Singh V , et al. What is the optimal imaging modality to detect endoleaks following elective endovascular repair of abdominal aortic aneurysm? Review Article 2018; ■■■: ■■■.

[8] Alamoudi A Haque S Srinivasan S , et al. Diagnostic efficacy value in terms of sensitivity and specificity of imaging modalities in detecting the abdominal aortic aneurysm: a systematic review. Int J Med Eng Inform 2015; 7: 15.

[9] Sommer WH Becker CR Haack M , et al. Time-resolved CT angiography for the detection and classification of endoleaks. Radiology 2012; 263(3): 917–926. 22623699 10.1148/radiol.12111217

[10] Hou K Zhu T Zhang W , et al. Dynamic volumetric computed tomography angiography is a preferred method for unclassified endoleaks by conventional computed tomography angiography after endovascular aortic repair. J Am Heart Assoc 2019; 8(8): e012011. 30957675 10.1161/JAHA.119.012011 PMC6507202

[11] Lehmkuhl L Andres C Lücke C , et al. Dynamic CT angiography after abdominal aortic endovascular aneurysm repair: influence of enhancement patterns and optimal bolus timing on endoleak detection. Radiology 2013; 268(3): 890–899. 23579050 10.1148/radiol.13120197

[12] Bent CL Jaskolka JD Lindsay TF , et al. The use of dynamic volumetric CT angiography (DV-CTA) for the characterization of endoleaks following fenestrated endovascular aortic aneurysm repair (f-EVAR). J Vasc Surg 2010; 51(1): 203–206. 19939612 10.1016/j.jvs.2009.07.101

[13] Apfaltrer G Lavra F Schoepf UJ , et al. Quantitative analysis of dynamic computed tomography angiography for the detection of endoleaks after abdominal aorta aneurysm endovascular repair: a feasibility study. PLOS ONE 2021; 16(1): e0245134. 33411747 10.1371/journal.pone.0245134 PMC7790279

[14] Koike Y Ishida K Hase S , et al. Dynamic volumetric CT angiography for the detection and classification of endoleaks: application of cine imaging using a 320-row CT scanner with 16-cm detectors. J Vasc Interv Radiol 2014; 25(8): 1172–1180.e1. 24837981 10.1016/j.jvir.2014.03.019

